# Evaluation of 3D ultrasound for image guidance

**DOI:** 10.1371/journal.pone.0229441

**Published:** 2020-03-26

**Authors:** David Iommi, Johann Hummel, Michael Lutz Figl

**Affiliations:** Center for Medical Physics and Biomedical Engineering, Medical University of Vienna, Vienna, Austria; University of Minnesota Twin Cities, UNITED STATES

## Abstract

**Purpose:**

In this paper we compared two different 3D ultrasound (US) modes (*3D free-hand mode and* 3D wobbler mode) to see which is more suitable to perform the 3D-US/3D-US registration for clinical guidance applications. The typical errors with respect to their impact on the final localization error were evaluated step by step.

**Methods:**

Multi-point target and Hand-eye calibration methods were used for 3D US calibration together with a newly designed multi-cone phantom. Pointer based and image based methods were used for 2D US calibration. The calibration target error was computed by using a different multi-cone phantom. An egg-shaped phantom was used as ground truth to compare distortions for both 3D modes along with the measurements of the volume. Finally, we compared 3D ultrasound images acquired by *3D wobbler mode* and *3D free-hand mode* with respect to their 3D-US/3D-US registration accuracy using both, phantom and patient data. A theoretical step by step error analysis was performed and compared to empirical data.

**Results:**

Target registration errors based on the calibration with the 3D Multi-point and 2D pointer/image method have been found to be comparable (∼1*mm*). They both outperformed the 3D Hand-eye method (error >2*mm*). Volume measurements with the *3D free-hand mode* were closest to the ground truth (around 6% error compared to 9% with the *3D wobbler mode*). Additional scans on phantoms showed a 3D-US/3D-US registration error below 1 mm for both, the *3D free-hand mode* and the *3D wobbler mode*, respectively. Results with patient data showed greater error with the *3D free-hand mode* (6.50*mm* − 13.37*mm*) than with the *3D wobbler mode* (2.99 ± 1.54 mm). All the measured errors were found to be in accordance to their theoretical upper bounds.

**Conclusion:**

While both 3D volume methods showed comparable results with respect to 3D-US/3D-US registration for phantom images, for patient data registrations the *3D wobbler mode* is superior to the *3D free-hand mode*. The effect of all error sources could be estimated by theoretical derivations.

## 1 Introduction

Three dimensional (3D) ultrasound (US) imaging is a promising approach for fast and non invasive visualisation in clinical environment. Compared to two dimensional (2D) images, the 3D presentation of the entire structure of an organ allows for a more intuitive orientation, repeatability of region of interest (ROI), and fusion with other 3D image modalities [1]. Unlike CT and magnetic resonance imaging (MRI) where the images are generally saved sequentially as a stack of parallel slices at a given orientation, ultrasound provides freely adjustable tomographic images in real-time. The orientation of the images is under the user control and therefore gives a high flexibility in applications. Apart from straightforward diagnostic visualization, US is used to support interventions such as image-guided surgery, ultrasound-guided radiotherapy planning, and image-guided biopsy [2, 3].

In this research study we compared two particular 3D-US reconstruction methods having in mind applications in image guidance [4, 5]. Our sample in-vivo images are abdominal prostate images because US-guided prostate therapy and US-guided prostate biopsy are among the most common applications for image guidance [6, 7, 8].

Guidance using real-time 3D US requires tracking the US image in 3D space. This is done by mounting a positional sensor (e.g. optical or electromagnetic sensor) to the US transducer and by computing the transformation from the image coordinate system to the coordinate system of an external position monitoring system (*US calibration)* [9].

*Spatial US calibration* for freehand 3D US using a conventional 2D US scan-head was first introduced by Detmer et al. [10]. As the 2D US transducer is swept over the volume, the position and orientation of the probe are recorded by an attached position sensor and a volume can be built by reformatting the US data, resulting in the so-called *3D free-hand mode* [1]. With the introduction of 3D US transducers based on a mechanically-swept transducer or *3D wobbler mode*, 3D US images can be acquired almost in real-time without the additional use of position sensors. This new generation of US devices creates volume data sets instead of 2D cross-sectional images. Furthermore, 3D US transducers with a 2 dimensional array of sensitive piezo elements are available [11]. Although these matrix arrays seem to have higher image quality the wobbler transducer are still more common in clinical routine.

The freehand method has advantages compared to the use of a 3D US transducer, such as lower cost and *larger* field of view (FOV) [1, 2, 12]. However, capturing a 3D US volume with free-hand techniques is difficult and sometimes cumbersome because of errors from the localization sensors, low accuracy of calibration, delays at each step of the reconstruction algorithm [13] and the demand of high-performance computing systems, such as graphical processing unit-based visualization [1].

In this work we compared *3D free-hand mode* and *3D wobbler mode* to see which is more suitable to perform the 3D-US/3D-US registration (transforming two separate 3D US images into the same coordinate system) for guidance systems in interventional surgery (e.g. patient repositioning, detection and correction of tissue deformation, etc. [4, 5]). 2D calibration methods were adopted using a tracking pointer and an N-wire phantom [14]. We also designed a new 3D-printed phantom to overcome shortcomings found with established methods for 3D calibration.

For both, the *3D free-hand mode and* 3D wobbler mode, we first calculated the common target registration error (TRE) [2, 15]. Then a volume reconstruction error was defined and calculated using a reference phantom as ground truth. In the second step we evaluated the dependence of the accuracy of the 3D-US/3D-US registration on the used 3D-US mode with phantom data and patient data from the prostate region.

Finally, the errors of each component of a typical transformation procedure were analyzed. The errors with respect to their impact on the final localization error were evaluated step by step theoretically and empirically.

## 2 Materials and methods

The *methods part of this section* is split up into three main parts: first we present two 3D *US* calibration methods and show how the calibration results can be applied to multiple depths of view. In the second part, two common 2D *US* calibration methods together with a 3D volume reconstruction are described. Finally, an error analysis is introduced.

### Specifications

A GE Voluson E6 ultrasound system with an RAB6-D convex transducer was used for 2D and 3D US imaging, respectively. The transducer, which works within a bandwidth of 2-8 MHz, consists of 192 piezo-electric elements. According to the manufacturer the US transducer had a resolution of axial 0.5 mm and lateral 2 mm.

The Polaris Spectra (NDI, Waterloo, Ca) optical tracking system (OTS) is assumed to provide a static accuracy of 0.25 to 0.35 mm within a volume of 1312 x 1566 x 1450 *mm*^3^ [16]. It enables accurate real-time 3D position and orientation tracking with tracking tools which are composed of passive marker spheres. To create our specific tracking tool such marker spheres were mounted on a 3D-printed frame made of polylactide (PLA). The resulting marker geometry was converted into standard tessellation language format (.stl). The fixture model was designed with the Rhinoceros 3D computer-aided design (CAD) software [17].

### US volume reconstruction methods

#### 3D free-hand mode

In order to reconstruct a 3D volume from 2D US images, each US image slice was inserted automatically by the PLUS software [18] into a 3D coordinate system which is defined by an OTS marker. Missing coordinates in the reconstructed volume were computed by weighted averages of nearby known voxels with a varying size spherical Gaussian kernel.

#### 3D wobbler mode

The GE Voluson E6 with the RAB6-D transducer was used in 3D mode. We used a volume angle of 85 degrees and maximum 2D size resulting in a field of view of 85x70 degrees with a 63.6 x 37.8 mm footprint. Data were stored as DICOM and loaded in the open source software platform 3D Slicer [19] to create the Cartesian image coordinate system.

### 2.1 3D Calibration methods

The frequency, depth of scanning, angle of sweeping and number of focal zones (one) of the transducer were the same for both 3D US calibration methods. For the 2D US calibrations, the same depth of scanning, frequency and focal zones were used. Different focal zones were used only in the volume reconstruction analysis.

#### 2.1.1 3D Multi-point target calibration

**Fig 1:**
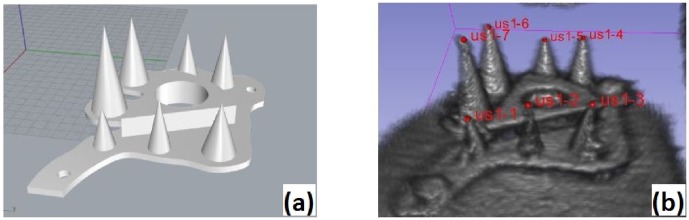
CAD model of the phantom for the 3D-US calibration (left) and its 3D ultrasound image on Slicer (right) with fiducials placed on the tips.

**Fig 2:**
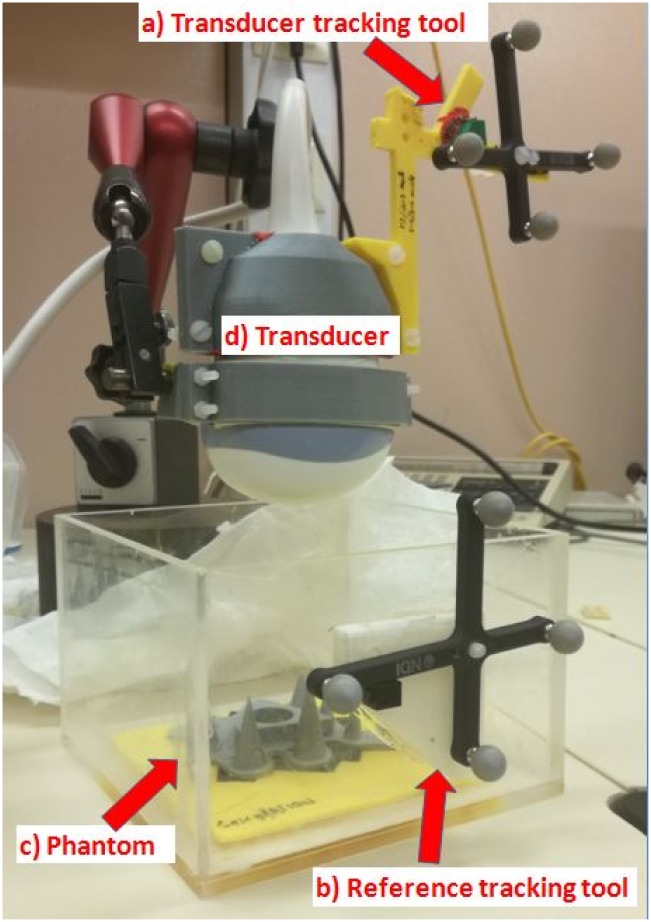
Setup for the transducer (d) calibration procedure: The phantom (c) was placed in a fixed position inside the water tank with a tracking tool attached on the tank (b) and on the transducer (a). The mechanical arm held the transducer in a constant position during image acquisition.

The phantom used for 3D calibration was made of polylactide (PLA) and consisted of a 5 mm thick base plate with cones placed on top of the plate where the tips of the cones served as fiducials (Fig 1(a)). The entire frame was placed in a tank made of perspex filled with water and fixed with screws (see Fig 2). A tracking tool was rigidly attached on the tank, representing the tank reference system.

The calibration procedure was based on multiple images of the frame while changing position and orientation of the transducer. The coordinates of the cone tips in the US image were determined by placing a cursor on each tip (Fig 1(b)) while the coordinates of the tips with respect to the tank reference system were determined using a calibrated stylus [20]. In order to minimize the jitter-error from the stylus measurements, each tip was measured 100 times while changing the camera positions 10 times. The distance between the phantom and the camera was always in the range of 1–1.5 m. Once the coordinates of the cone tips (i.e. the fiducials) were known in both coordinate systems a point-to-point registration [21] was conducted.

To increase accuracy we introduced an additional *phantom calibration*. The coordinates of the cone tips were gathered in the coordinate system of the tank reference system (*P*_*Reference*_) as well as in the coordinate system of the CAD model of the phantom (*P*_*Phantom*_). This resulted in a transformation *T*_*Ph*→*Ref*_ which was used to re-calculate the coordinates of the fiducials in the reference system from the more precisely measurable points in the CAD volume.

The calibration matrix *T*_*US*→*Transducer*_, which expresses the transformation between the US image coordinate system and the coordinate system of the optical marker attached to the transducer, was finally obtained applying the following equation:

$$\begin{array}{c} T_{Ref\to Transducer}\times T_{Ph\to Ref}\times P_{Phantom}=T_{US\to Transducer}\times P_{US} \end{array}$$ (1)

where *T*_*Ref*→*Transducer*_ is the transform between the reference and the transducer tracking tool given by the OTS. The tracker was connected to the 3D Slicer via PLUS toolkit (Public software Library for US imaging research) [19].

To accomplish a 3D-US calibration, 15 images were taken, with the transducer moved between image acquisitions to realize different viewing angles. The transducer was mounted on a flexible arm to avoid motion artifacts in the recorded tracker data. To define reproducible imaging conditions the measurement series was started with the transducer and the box tracking sensors pointing directly to the optical camera. For subsequent scans the US probe was rotated from +35° to −35° around its principal axis covering 15 orientations relative to the tank reference system. In total, ninety fiducials were collected for the point-to-point registration.

#### 2.1.2 3D Hand-eye calibration

The Hand-eye method [22, 23] can be applied to compute the calibration matrix *T*_*US*→*Transducer*_ by using transformation matrices *T*_*US*(*i*)→*US*(*j*)_ from different volumes *US*_*i*_, *US*_*j*_ taken from the phantom from different perspectives [24]. These transformations can be expressed by:

$$\begin{array}{c} T_{US(i)\to US(j)}=T_{US(i)\to Transducer(i)}\times T_{Transducer(i)\to Ref}\\ \times T_{Ref\to Transducer(j)}\times T_{Transducer(j)\to US(j)} \end{array}$$ (2)

**Fig 3:**
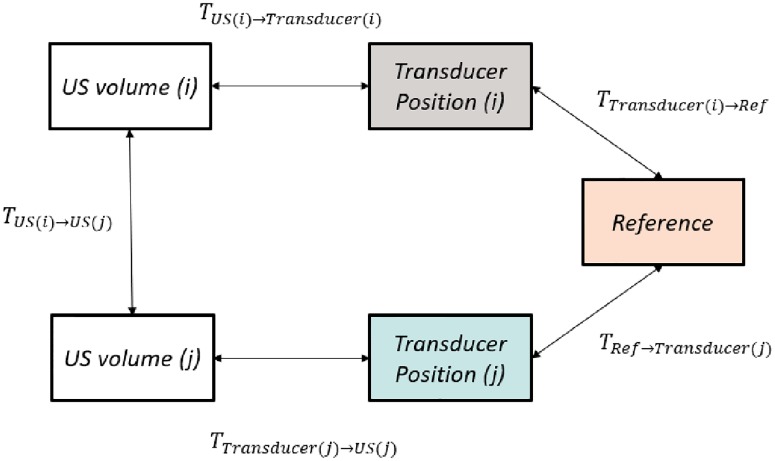
Hand-eye US calibration loop: The transformation between arbitrary US volumes *US*_*i*_ and *US*_*j*_ can be found indirectly via the tracked sensor positions or directly via 3D intra-modality image registration.

*T*_*US*(*i*)→*Transducer*(*i*)_ (and also *T*_*Transducer*(*j*)→*US*(*j*)_, respectively) represents the calibration matrix. *T*_*Transducer*(*i*)→*Ref*_ (and *T*_*Ref*→*Transducer*(*j*)_, respectively) is given by the OTS as the transform between the tracking tool and the tracker camera. The transforms *T*_*US*(*i*)→*US*(*j*)_ were determined using 3D Slicer by registering arbitrary US volumes *US*_*i*_ and *US*_*j*_ taken at the transducer positions *i* and *j*, respectively. Fig 3 gives a graphical illustration of formula (2).

Right-multiplying Eq (2) by $T_{Transducer\to US}^{-1}$ (3) leads to

$$\begin{array}{c} T_{US(i)\to US(j)}\times T_{US\to Transducer}=T_{US\to Transducer}\times T_{Transducer(i)\to Ref}\times T_{Ref\to Transducer(j)}. \end{array}$$ (3)

This equation has the form:

$$\begin{array}{c} AX=XB \end{array}$$ (4)

where A = *T*_*US*(*i*)→*US*(*j*)_, B = *T*_*Transducer*(*i*)→*Ref*_×*T*_*Ref*→*Transducer*(*j*)_, X = *T*_*US*→*Transducer*_.

To solve this equation system unit quaternions were used to compute the rotation component in closed form. Four different metrics were tested for this task: Horaud [28], Tsai [23], Park [25] and Liang [26]. The same phantom US images used for the Multi-point calibration were also used for the Hand-eye calibration procedure.

#### 2.1.3 3D mode calibration at multiple depths

The Multi-target and Hand-eye calibrations for the *3D mode* were performed at 7.4 cm depth to obtain an optimal trade-off of lateral resolution and distribution of the fiducials. Subsequently, the calibration matrices for greater depths were derived from this reference calibration matrix *T*_*US*→*Transducer*Â (7.4*cm*)_. This was done by scanning the phantom at multiple depths, keeping the transducer fixed. The new volumes were then registered with the reference volume to obtain the transforms $T_{V_{\left(7.4cm\right)}\to V_{NewDepths}}$. According to Eq (5) the new calibration matrices can be calculated as:

$$\begin{array}{c} T_{US\to Transducer\;(NewDepth)}={\left(T_{US\to Transducer\;(7.4cm)}\right)}^{-1}\times T_{V_{\left(7.4cm\right)}\to V_{NewDepth}} \end{array}$$ (5)

**Fig 4:**
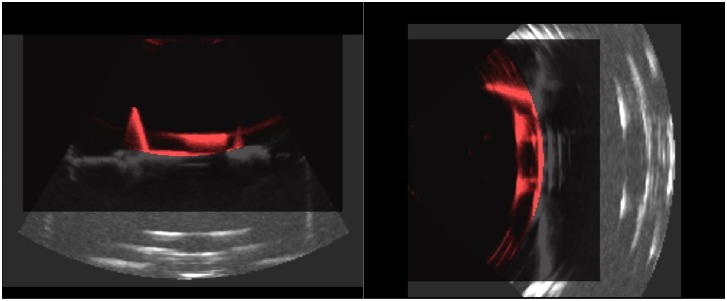
The 3D volume at 7.4 cm (red) is a sub-set of the volume with 15 cm scan depth (grey). They are registered to obtain the transform *T*_7.4*cm*→15*cm*_. Then the calibration matrix at 15 cm depth can be calculated. Thus, this transformation is a simple translation.

For the GE Voluson E6, the saved images have different three dimensional sizes (x,y,z) depending on the scan settings (depth, beam width etc.). By uploading them on 3D Slicer, a new origin of image coordinate system is generated automatically. No scaling operation on the same object is performed by the machine while using different settings. The consequence is that these volumes can be overlaid on each other with a rigid translation (Fig 4). Therefore, $T_{V_{\left(7.4cm\right)}\to V_{NewDepths}}$ represents a translation.

### 2.2 2D US calibrations and volume reformatting

#### 2.2.1 2D US pointer calibration

For 2D calibration, the 3D Slicer—PLUS toolkit was implemented and the *tracked US calibration* as described in the 3D Slicer Tutorial was conducted [19]. For this purpose the US transducer was mounted in a fixed position upon an empty water tank and the calibrated NDI pointer was swept within the US image FOV. Tracker data were recorded automatically and the corresponding image coordinates were determined by marking at the maximum intensity profile of the pointer tip on the image for certain time points. This results in a list of fiducials given in two coordinate systems i.e. the image and the OTS coordinate system. Finally, the calibration matrix was calculated by means of a point-to-point registration. This procedure was performed twice to get the calibration matrices for 7.4 cm and 15 cm scan depths.

#### 2.2.2 D US N-wire calibration

**Fig 5:**
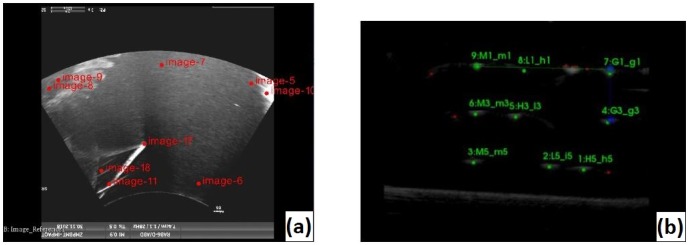
2D spatial US calibration techniques: By applying the pointer method, fiducials were collected on the image by looking at the maximum intensity profile of the pointer tip on the image (Fig **5(a)**). N-shaped wires are seen as dots on the images which are detected and segmented by the PLUS software. The result of the segmentation can be identified as the green cursors (see Fig **5(b)**).

An N-shaped wire was mounted in a water filled plastic tank and the PLUS algorithm from 3D Slicer was used. This algorithm computes the transformation between the phantom object coordinate system and the coordinate system of the tracking tool attached to the transducer by point matching (Fig 5(b)). In particular, the ultrasound scan of an N-shaped wire results in images with white dots representing the intersection of the N-shaped wires with the US scanning plane. Each dot was segmented automatically and the coordinates of the dots in the OTS coordinate system were computed by means of geometric triangulation. Next, a point-to-point registration was applied to receive the calibration matrix. The N-shaped wire phantom used was designed for 9 cm depth at the most. For this reason, this method could not be used at greater depths.

### 2.3 Error analysis

To evaluate the 2D/3D US calibration methods the TRE was analyzed at the scanning depth of 7.4 cm and 15 cm. Secondly, an error analysis on the volume reconstruction from 2D and 3D ultrasound modalities was performed. Since the acquired 2D images which were used to generate a 3D image were arranged as a fan, the distance between the acquired 2D US images increases with increasing axial distance. Differences in the volume reconstruction might also appear because the *3D wobbler mode and the* 3D freehand mode *use different interpolation techniques*. Therefore, the US volume reconstruction might be affected by an error which compromises the accuracy of the registration. To investigate the degree of volume distortion depending on the method, a given volume was compared with the reconstructed volumes found in the 3D-images. Finally, the 3D-3D US/US registration was tested for the *3D freehand mode* and the *3D mode* by computing a metric error from a rigid registration.

#### 2.3.1 US calibration error analysis

**Fig 6:**
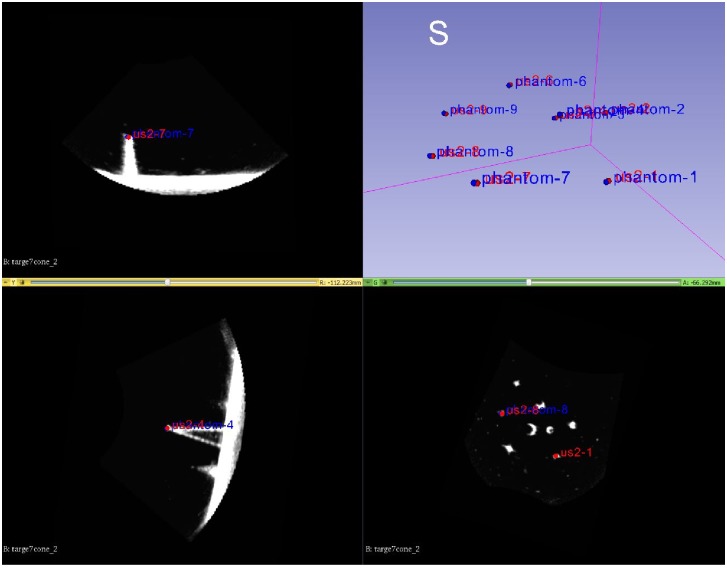
US target registration error on 3D Slicer: Points are collected on the image (red dots) and overlaid by applying the transform to the ground truth phantom coordinates (blue dots).

The fiducial registration error (FRE) was calculated for the *3D wobbler mode* calibration and for the 2D calibration methods. For both 3D modes an additional PLA phantom featuring cones in a new configuration was used to compute the TRE. The phantom calibration was applied as described in section 2.1.1 and the euclidean distance between the resulting reference points and transformed ultrasound image points (Fig 6) was calculated as the norm of the difference vector *T*_*Diff*_ given in Eq 6. Eighty fiducials from ten images were used to compute the TRE for each method.

$$\begin{array}{c} T_{Diff}=\left(T_{Ph\to Ref}\times P_{Ph}\right)-\left(T_{Transducer\to Ref}\times T_{Us\to Transducer}\times P_{Us}\right) \end{array}$$ (6)

To evaluate the impact of the number of US images used for calibration on the TRE, the calibration matrix was computed with an increasing number of images averaging over permutations as described in [24]. With regard to the Multi-target method, the matrix was computed starting with one single image; the Hand-eye method instead was run with four starting images to obtain consistent results. The images were added one by one and the TRE was calculated at each stage.

#### 2.3.2 Volume error analysis

**Fig 7:**
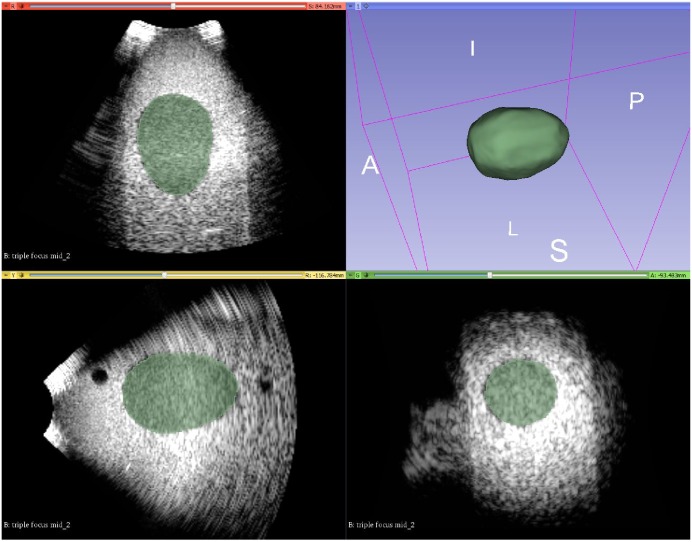
Example 3D mode US image of the egg-phantom in 3D Slicer: The egg-phantom was segmented and the software returned the segmentation result (green) in the xyz planes and in the 3D volume rendering (right top of the image).

The CIRS tissue-mimicking phantom Model 560H [27] was used to evaluate the imaging system performance by 3D volumetric measurements [27]. The phantom combines monofilament line targets, six non-echogenic cylindrical targets of varying sizes and an egg-shaped target structure. The target egg volume was 91.6 *cm*^3^. The 3D images of the egg acquired with the *3D wobbler mode* and the *3D free-hand mode* were segmented with control points: for all three projections, the observer moved a loop of interconnected points (around thirty per projection) on the contour of the surface. The final volume was calculated by least square sphere fitting on 3D Slicer (Fig 7), similar to Fagerquist et al. and Uittenbogarrd et al. [28, 29] Three different depths were investigated. This was done by increasing the distance between the bottom surface of the egg phantom and the transducer. Furthermore, the egg phantom was scanned using one focal zone and three focal zones, respectively. Three focal zones are not used for the largest depth because it wasn’t possible to set the beam in a way to cover the entire object. A total of five acquisitions were taken and the mean value and the difference (%) from the ground truth volume were calculated.

#### 2.3.3 Evaluation of 3D-US modes for 3D-US/3D-US registration

To evaluate the dependence of the accuracy of a 3D-US/3D-US registration on the used 3D-US mode, a registration error was determined using egg phantom. The OTS was not moved during this procedure. Two tests were performed:

- In a first image acquisition series the transducer was positioned always in the same orientation (the straight position of the transducer in front of the camera). Between the individual images, the transducer was lifted and applied again. The first image served as the reference image and was registered to the others.
- The next series of images was taken with different angles, as listed in the appropriate tables in the results section and registered to the reference image. This was done to evaluate how the orientation of the transducer affects the volume reconstruction and the accuracy of the registration.

**Fig 8:**
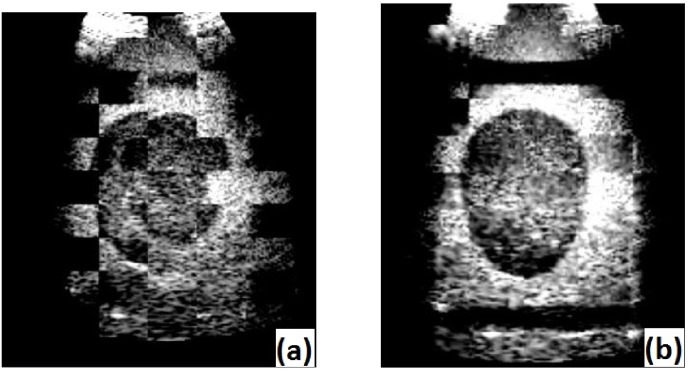
Checkerboard image of egg phantom images with 3D *Slicer*. On the left hand side, the unaligned images can be seen, on the right hand side the images after alignment using the US calibration are overlaid.

**Fig 9:**
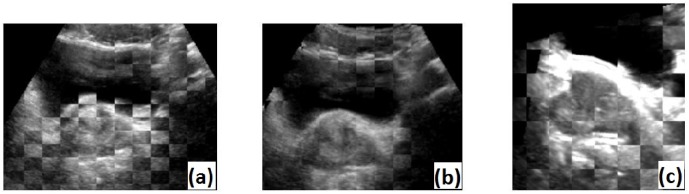
**Checkerboard image of prostate images with 3D Slicer: (a) shows the the unaligned images and (b) the images after alignment using the US calibration using the *3D wobbler mode*.** In (c) the *3D free-hand* reconstructed volumes were aligned.

The images were organized in the hierarchy representation on 3D Slicer which automatically transforms multiple ultrasound volumes into the same reference system (i.e. of the attached tracking tool), see Figs 8 and 9. A region of interest was considered and the Mattes mutual information metric (MI) [30] registration was applied. In an error less procedure the result of registration should be the unit matrix, assuming a perfect calibration, otherwise it gives an indicator of the registration error (*E*_*error*_). Therefore, the Frobenius-norm of this matrix was used as a measure of the accuracy of image registration. Additionally, *E*_*error*_ was calculated as the norm of the distance between the unit vector and the unit vector multiplied with the resulting registration matrix as described in [5].

The same procedure as above was repeated to evaluate images of a prostate. The transducer was moved by the user between two different recordings. Like described above, the *E*_*error*_ was calculated as the norm of the distance between the unit vector and the unit vector multiplied with the resulting registration matrix.

#### 2.3.4 Overall TRE in a typical application

**Fig 10:**
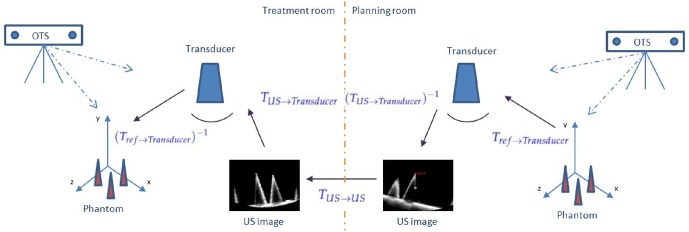
Full transformation chain from the planning to the treatment room.

Fig 10 shows a sketch of the full transformation chain of a typical guidance system in tele-therapy or biopsy [4, 8, 31]. The purpose of such a system is to align pre-operative data to (real-time) images in the treatment room.

The markers attached to the phantom/patient and to the US transducer defined two reference coordinate systems (*ref*). The arrows in Fig 10 indicate the necessary transformations to register a point in the planning room (*P*_*p*_) with the corresponding point in the treatment room (*P*_*t*_). Eq 7 shows the corresponding matrix equation:

$$\begin{array}{c} P_{t}={\left(T_{ref\to Transducer}\right)}^{-1}\times T_{US\to Transducer}\times T_{US\to US}\times \\ \times {\left(T_{US\to Transducer}\right)}^{-1}\times T_{ref\to Transducer}\times P_{p} \end{array}$$ (7)

where *T*_*ref*→*Transducer*_ transforms the phantom/patient reference coordinate system to the transducer coordinate system. *T*_*US*→*Transducer*_ is the US calibration matrix and *T*_*US*→*US*_ is the rigid transformation between the 3D-US images taken in the planning and treatment room, respectively.

We used the multi-cone phantom for testing this transformation chain. To simulate two different operating rooms, we moved both the OTS and the US transducer between the two recordings of the US image. The phantom was not moved during the procedure. Therefore, the expected repositioning matrix should be the unit matrix. This test was done with the 3D wobbler mode as it provides all the components of the transformation chain. A total of eight images were acquired. The first image had the transducer in straight position in front of the camera and was used as a reference image. The other images were taken with different angles/orientations and registered to the reference image. The TRE was calculated based on the coordinates of the cones tips from the phantom.

#### 2.3.5 Evaluation of the effects of error sources

To give an idea how the individual pieces of such a guidance system contribute to the overall TRE, the errors from the different components of the transformation chain (see Eq 7 were analyzed theoretically and compared to measurements.

The theoretical error analysis was based on [32]. The effect to the TRE of a purely translational error *t* that appears in only one matrix in Eq 7, which e.g. could be the case for *T*_*US*→*US*_, is:

$$\begin{array}{c} TRE_{\Delta }\leq ||t||. \end{array}$$ (8)

For a purely translational error *t* in two matrices which could be the case for *T*_*ref*→*Transducer*_ and *T*_*US*→*Transducer*_, the effect to the overall error is:

$$\begin{array}{c} TRE_{\Delta }\leq 2||t||. \end{array}$$ (9)

The complete theoretical derivation of Eqs 8 and 9 can be found in the Appendix. To evaluate the influence of the tracking system and the US device, the image registration step was eliminated by keeping the phantom in a steady position.

*Error from the tracking system*. For a mainly translational perturbation *t* on *T*_*ref*→*Transducer*_, *TRE*_Δ_ was estimated according Eq 9. To evaluate the empirical error caused by the OTS, the two different rooms were simulated by moving the OTS to five different positions, generating five ${\tilde{T}}_{ref\to Transducer}$ matrices. Since the multi-cone phantom was not moved during this procedure (*P*_*t*_ = *P*_*p*_), the expected repositioning matrix should be the unit matrix. The tracked transducer was fixed on a rigid mount and therefore *T*_*US*→*US*_ also simplified to the unit matrix. Then the *T*_*US*→*Transducer*_ and its inverse also multiply theoretically to the identity. Thus, the resulting registration matrix only includes errors from the OTS (*Err*_*tracking*_) should be the unit matrix:

$$\begin{array}{c} Err_{tracking}={\left(T_{ref\to Transducer}\right)}^{-1}\times {\tilde{T}}_{ref\to Transducer} \end{array}$$ (10)

The TRE was calculated based on the coordinates of the cones tips from the phantom and compared with its theoretical estimation:

$$\begin{array}{c} TRE_{tracking}=mean||P_{t}-\left(Err_{tracking}\times P_{p}\right)\left|\right| \end{array}$$ (11)

This whole procedure was repeated with different angles/positions of the transducer relative to the reference coordinate system of the phantom.

*Error resulting from the US image resolution*. The lateral and axial resolutions were measured with the monofilament line targets of the US phantom Model 560H [27]. As the FRE can be approximated by the fiducial location error (FLE) for a large number of fiducials and the FLE is in the magnitude of the resolution of the US images, the FRE is determined by the (lateral) resolution [33, 34, 35]. In the N-wire phantom the localization is replaced by a fitting process minimizing the lateral (squared) error.

*Error from the US calibration matrix*. Adding a purely translational perturbation *t* on the *T*_*US*→*Transducer*_ results in an additional *TRE*_*cal*_Δ. This error can be constrained by Eq 9. The applied perturbation *t* was based on the TRE from the US calibration from section 3.1. To measure this error empirically, the US calibration matrix was calculated using the Multi-target method. We acquired five phantom images: the first image was used as reference and the other images were taken with different angles/positions and registered to the reference image. The phantom and the OTS were not moved in this procedure. The TRE was computed as described in section “Evaluation of 3D-US mode for 3D-US/3D-US registration”. The purely translational perturbation *t*(2/1/0), ||*t*|| = 2.23 mm was then introduced. The empirical deviations (*TRE*_*cal*_Δ) from the previous TRE were calculated.

*Error from US-US registration*. A purely translational perturbation *t*(2/2/1) with ||*t*|| = 3*mm* as found in literature [5] was introduced to *T*_*US*→*US*_. A total of eight images were acquired. Similarly to section 2.3.4, the first image was used as a reference image. The other images were taken with different angles/orientations and registered to the reference image. We moved both the OTS and the US transducer between the recordings of the US images. The empirical deviations (*TRE*_*reg*_Δ) from the previous TREs were calculated for all measurements, after introducing the perturbation to the *T*_*US*→*US*_ matrix.

## 3 Results

### 3.1 Calibration error analysis

Table 1 shows the FREs for the *3D wobbler mode* calibration and the 2D calibrations. With the Multitarget point method a FRE of 0.87 mm was achieved. Using the N-wire phantom for 2D calibration, the FRE was 0.40 mm at 7.4 cm depth while the FRE amounted to 0.52 mm applying the 2D US pointer method.

**Table 1:** Fiducial registration error for the 3D and 2D calibration techniques.

FRE (Fiducial registration error) (mm)		
Scan depth	7.4 cm	15 cm
**3D wobbler mode**		
Multitarget point	0.87	/
**2D calibrations**		
Pointer method	0.52	0.43
N-wire phantom	0.40	/

Table 2 shows the TRE for the *3D wobbler mode* and *3D free-hand mode*. The mean TRE for the 3D Multi-target point was 1.08 mm for a scan depth equal to 7.4 cm and 1.25 mm for 15cm depth. The best result was found for the *3D free-hand mode*: based on the 2D N-wire method the TRE was 0.69 mm. The 2D pointer based method (1.00 mm) was comparable to *3D wobbler mode*. Based on the Hand-eye calibration, the TRE was higher than 2 mm for all applied metrics.

**Table 2:** Target registration error for the 3D and 2D calibration techniques.

TRE (Target registration error) (mm)		
Scan depth	7.4 cm	15 cm
**3D wobbler mode**		
Multitarget point	1.08±0.48	1.25±0.46
Hand-eye method		
*Horaud*	2.23±0.47	2.44±0.48
*Tsai*	2.03±0.55	2.49±0.42
*Park*	2.22±0.45	2.37±0.42
*Liang*	2.22±0.45	2.39±0.46
**3D free-hand mode**		
Pointer method	1.00±0.85	1.12±0.57
N-wire phantom	0.69±0.37	/

**Fig 11:**
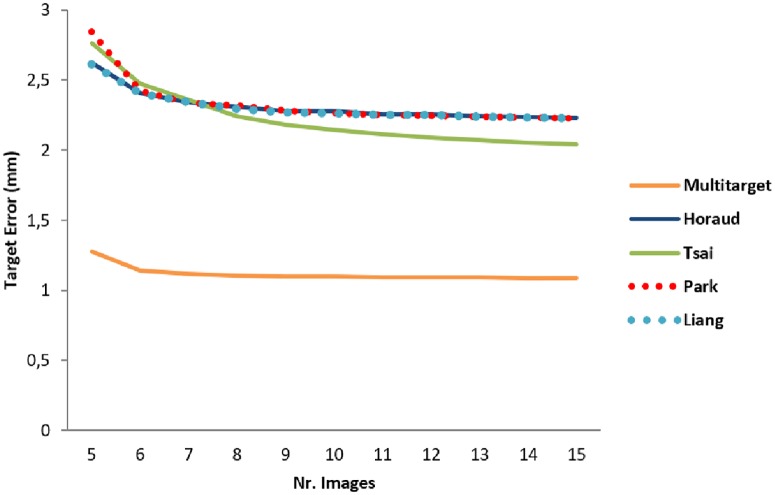
Target registration error trend at the scanning depth of 7.4 cm. The Multi-target (orange line) showed the lowest TRE of all 3D mode calibration methods. By adding more calibration images, the error did not improve markedly. With the Hand-eye method, the error decreased for the first five image samples but it was generally higher compared to the Multi-target method.

**Fig 12:**
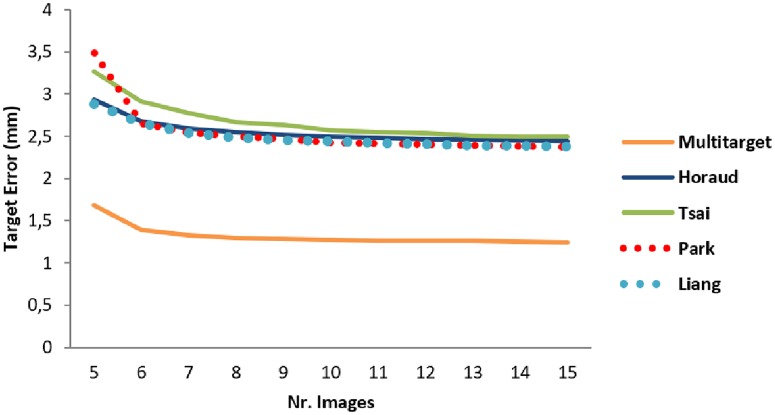
Target registration error trend at the scanning depth of 15 cm. Like the previous measurements, the Multi-target method (orange line) had target error lower than the Hand-eye.

As described in section **1.3.1**, the impact of the number of images used to compute the calibration matrix for the 3D calibration was evaluated. The results are shown in Figs 11 and 12 for the scanning depths of 7.4 cm and 15 cm, respectively. The Multi-target method resulted in a TRE less than 2 mm with just one image. The error decreased slightly until a minimum was reached with about five images. With the Hand-eye method, the error decreased after five samples but was always higher compared to the Multi-target method.

### 3.2 Volume error analysis

Table 3 shows that the *3D free-hand* volume results were overall the closest to the ground truth. In fact, with one focal zone, the reconstructed volumes differed from the actual volume by less than 6% for all for all three distances analyzed. By using three focal zones, as shown in Table 3, the result did not change at the closest scan distance, but improved for the mid one (3.11%). The *3D wobbler mode* offered slightly better results with one focal zone (5.60%), but was worsened by increasing the distance of the object from the transducer aperture (9.23% and 11.61% for the middle and further scan distance respectively). With three focal zones, the error decreased for the middle range distance, but was still outperformed by the *3D free-hand mode*.

**Table 3:** Mean value and deviations of the egg phantom volumes for the two US modalities from the reference value of 91.6 *cm*^3^. With only one focal zone, the focus was placed manually at the level of the egg phantom center of mass. With three focal zones, the focus was set to cover entirely the width of the egg phantom. The distances listed refer to the ones between the bottom of the egg phantom surface and the transducer aperture.

**Phantom reconstruction error /one focal zone:**		
**Close distance (82.62 mm)**	Mean(*cm*^3^)	Dev (%).
*3D wobbler mode*	97.71±0.24	5.60
*3D free-hand mode*	98.45±2.37	6.27
**Middle distance (112.15 mm)**		
*3D wobbler mode*	101.67±1.42	9.23
*3D free-hand mode*	97.71±1.53	5.59
**Further distance (175.88 mm)**		
*3D wobbler mode*	104.28±2.53	11.61
*3D free-hand mode*	97.92±4.10	5.79
**Phantom reconstruction error /three focal zones:**		
**Close distance (82.62 mm)**	Mean(*cm*^3^)	Dev(%).
*3D wobbler mode*	99.59±0.98	8.73
*3D free-hand mode*	97.38±1.52	6.31
**Middle distance (112.15 mm)**		
*3D wobbler mode*	98.27±2.12	7.28
*3D free-hand mode*	94.45±0.81	3.11

### 3.3 Evaluation of 3D-US mode for 3D-US/3D-US registration

#### Egg-shape phantom

By keeping the transducer in a fixed position, the mean registration *E*_*error*_ resulting from five registrations was found to be 0.57 mm for the *3D free-hand mode* (Frobenius norm of the error matrix: 2.04) and 0.70 mm for the *3D wobbler mode* (Frobenius norm of the error matrix: 2.10), respectively.

Table 4 shows the registration errors resulting from different transducer orientations.

**Table 4:** The registration error for the *3D free-hand mode* and the *3D wobbler mode*. Orientations/angles (degree) are relative to the axis of reference position of the transducer, straight in front of the camera.

**3D-US/3D-US free-hand mode**					
Nr.	*Error (mm)*	*Frobenius*	*x°*	*y°*	*z°*
1	3.71	4.21	-4.03	-8.35	-19.31
2	1.05	2.26	-2.71	-7.82	-13.21
3	0.92	2.20	-0.47	17.89	-4.36
4	1.38	2.43	-14.93	-5.54	-0.24
5	0.73	2.13	-2.58	19.43	0.21
6	1.82	2.70	7.05	-37.34	33.19
**3D-US/3D-US wobbler mode**					
Nr.	*Error (mm)*	*Frobenius*	*x°*	*y°*	*z°*
1	0.61	2.09	-0.81	-4.86	-13.61
2	0.61	2.08	1.63	4.67	22.11
3	0.49	2.06	-0.03	24.71	-1.55
4	0.51	2.06	1.85	40.11	-2.40
5	1.61	2.56	-11.82	-1.36	1.13
6	1.07	2.28	5.25	-32.12	28.39

#### Prostate 3D-US/3D-US registration

The errors for the *3D free-hand mode* were between 6.50 mm (minimum) and 13.37 mm (maximum). The *3D wobbler mode* showed much better results (see Table 5) with a mean error of 2.67±1.46 mm (0.84 mm and 4.73 mm as minimum and maximum values, respectively).

**Table 5:** Registration error arising with patient data for the *3D wobbler mode*. Orientations/angles (degrees) are relative to the axes of the reference position of the transducer given with the first reference scan.

3D-US/3D-US wobbler mode					
Nr.	*Error (mm)*	*Frobenius*	*x°*	*y°*	*z°*
1	2.56	3.22	0.38	-4.25	-1.91
2	0.84	2.17	5.90	-7.77	-1.01
3	2.81	3.44	2.13	-1.66	1.79
4	3.71	4.21	-11.82	-1.36	1.13
5	1.27	2.37	0.32	-0.03	0.08
6	4.73	5.12	2.94	-2.21	3.30

### 3.4 Overall TRE in a typical application

By computing the overall TRE of the full transformation chain, as described in section **2.3.4**, we found an average TRE of 1.87 mm.

### 3.5 Evaluation of the effects of error sources

#### Error from the tracking system

Table 6 shows the *TRE*_*tracker*_ and an upper bound according to Eq 9. This experiment was repeated five times whereby the relative orientation between the tools on the transducer and the phantom was varied (last three columns).

**Table 6:** *TRE*_*tracker*_ and theoretical upper bound. Rotation of the transducer relative to the reference tool.

Nr.	*TRE*_*tracking*_	2||*t*||	*x°*	*y°*	*z°*
1	0.56	0.89	13.73	1.00	1.31
2	0.86	0.95	8.05	27.22	4.09
3	0.83	1.27	37.21	-1.07	2.98
4	1.66	2.29	16.74	-36.46	-20.10
5	1.06	1.97	11.03	-0.03	-21.32

#### Error from US image resolution

For the imaging parameters used throughout the paper, the measured axial resolution was found to be 0.5 mm, while the lateral resolution was 2 mm. These numbers are similar to the manufacturer’s specifications.

#### Error from the US calibration matrix

Table 7 shows the additional introduced error *TRE*_*cal*_Δ caused by a translational deviation *t* to the US calibration matrix. The data refer to four cases with different angles/orientations of the US transducer respect the first scan (reference scan).

**Table 7:** Additional error *TRE*_*cal*_Δ introduced by the perturbation *t* and upper bound. Orientations/angles (degree) of the transducer between the first scan and the other scans.

Nr.	*TRE*_*cal*_Δ	2||*t*||	*x°*	*y°*	*z°*
1	0.28	4.46	19.19	-0.89	1.01
2	0.55	4.46	-17.71	-4.15	3.74
3	0.78	4.46	-5.22	-1.45	-16.59
4	0.30	4.46	2.54	-22.45	16.7

#### Error from US-US registration

The average *TRE*_*reg*Δ_ resulting from the perturbation (||*t*|| = 3.0) applied to *T*_*US*→*US*_ was 2.67 mm.

## 4 Discussion

In this work we compared two 3D US modes to evaluate which one is more suitable to perform a 3D-US/3D-US registration of the prostate. The evaluation criteria included the analysis of the target error, the volume reconstruction error and the registration error with phantoms and patient data from the prostate region. As the required US calibrations affect the registration success considerably, we adopted different calibration methods for 2D and 3D modalities and compared them in terms of the target error achieved. In the course of 3D calibration, we introduced a new 3D printed phantom which increases accuracy and usability. Secondly, the volume reconstruction accuracy was analyzed because volume distortions could affect the accuracy of the registration and the understanding of the full 3D spatial anatomy by the physician. Over all, the 3D-US/3D-US registrations with phantom and patient data have shown that *3D wobbler mode* resulted in lower registration errors than the *3D free-hand mode*.

With respect to calibration, the *3D free-hand mode* has shown the lowest error from phantom measurements: we obtained a TRE of 0.69±0.37 mm using the N-wire method, comparable to the 1.0 ± 0.12 mm point reconstruction accuracy found in [19]. The pointer method also resulted in a low TRE (1.00±0.85 mm, see Table 2). The 3D Multi-target calibration was less precise (the maximum error amounted to 1.26±0.45 mm, Table 2) but was comparable to the free-hand techniques. The 3D Hand-eye procedure showed the worst result, comparable with the previous work from Schlosser et al. [24] (2.4 mm error). The results showed also that exploring using multiple acquisition angles improves the precision of the calibration. As shown in Fig 11 the Multi-target TRE decreased from 1.26 mm to 1.12 mm by applying three more images. Additional images did not improve the TRE considerably. In Shinya et al. [36] a TRE of 2.2 mm was reached with eight images but no evaluation was made on the relationship between the TRE and the numbers of applied images. In Vasconcelos et al. [37] a TRE of 2.39 mm was obtained using around 10 acquisitions. The Hand-eye method results showed no considerable improvement after applying more than five images for the calibration matrix computation which is comparable to the results found by Schlosser et al. [24].

In terms of feasibility of the procedures, the calibration with the N-wire phantom is the most convenient. It takes a few seconds, is robust and independent from the user experience [38]. Without the availability of a phantom, the alternative 2D pointer method requires only a calibrated pointer tip but is not reproducible. In fact, the user has to steadily move the pointer tip, aligned with the scan plane, and carefully select the fiducials on the image at certain time points. This requires the user to have certain skills and experience [38]. The manual selection of fiducials is simplified by the use of a phantom: with our 3D-printed phantom, introduced in this work, it was possible to collect fiducials from a few images in the 3D multi-target method resulting in a low target error. A similar printed phantom was used for the Hand-eye method; although the procedure had the advantage of not manually selecting fiducials, it showed the worst calibration results.

The volume reconstruction showed the lowest error for the *3D free-hand mode*. The measurements performed at different depths yielded an error range of 3.11%±6.27% (Table 3). The deviation from the volume ground truth obtained was comparable with the results from Fenster et al. [15] (5.7% and 4.4% for the 10 cm and 15 cm depth settings, respectively). The *3D wobbler mode* volume was further from the ground truth with an error of 5.60%±11.61% (Table 3). Increasing the number of focal zones yielded a lower error of 7% for the *3D wobbler mode* and 3% for the *3D free-hand mode*, while with one focal zone the errors were around 9% and 6%, respectively. Nevertheless, using just one focal zone showed a better result at lower depths (Table 3).

The acquisition of the 2D images for the *3D free-hand mode* proved to be an obstacle for this method: the user had to keep the line of scanning and had to move the transducer slowly to obtain enough slices for the reconstruction algorithm. This is important especially for greater scan depth. A possible solution could be found in a handheld motorized scanning device with a mechanical system performing a linear or tilted scanning motion, as in Fenster et al. [15]. The *3D wobbler mode* does not have this problem: the transducer’s sensor array can scan automatically the whole volume, while maintaining the transducer in a fixed position. The scan time is generally comparable with the two modes (around 6-9 seconds). For the *3D wobbler mode*, it depends on the settings selected (with deep scans at low frequency mode the wobble transducer arrays take more time to sweep over the volume).

The tests for the evaluation of 3D-US mode for 3D-US/3D-US registration yielded opposing results: scans on phantoms showed an error of approximately 1 mm with both modalities (Table 4), while for patient data the highest errors occurred with the *3D free-hand mode* where all tests indicate an error in the range of [6.50 mm—13.37 mm]. A mean error of 2.67±1.46 mm was found for the *3D wobbler mode* (Table 5), outperforming the free-hand mode significantly. The registration results for the *3D wobbler mode* were comparable with Kaar et al. [5] (2.99 ± 1.54 mm). This error is within the level of tolerance for prostate repositioning in tele-therapy and in the error range of successful prostate biopsies. ([6] mentions 4mm for repositioning, [8] measures around 3 mm for biopsies). Therefore, the *3D wobbler mode* US could perform repositioning and registration within the error tolerance level.

Tissue deformation could be a major issue for real patient image registrations: the pressure and the point of origin of the transducer deform the tissues in different ways for different scans. They compromise the volume reconstruction, leading to a poor registration result. Because of the deformations, it is difficult to acquire enough US images with the free-hand mode on the same line of scanning. Another advantage of the *3D mode* is that the user does not need to sweep over the region of interest: the array automatically reconstructs the volume. Hence, many movement artifacts are suppressed here, resulting in a better image quality. The user just needs to hold the transducer steady, without changing the pressure on the subject, to limit the deformations and get an accurate positioning of the image by the OTS.

The registration error is also affected by artifacts, such as acoustic shadowing, refraction, side lobes etc., which impair image quality considerably. Particularly with increasing depth, lateral resolution decreases and the object is distorted depending on the scan direction. This applies to both the *3D free-hand mode*, where each 2D slice is interpolated after sweeping the transducer, and the *3D wobbler mode*, where the array of sensors is automatically shifted. Therefore, with rigid registration, a mismatch was found between the outer surfaces in both the egg phantom and the prostate images. Despite this, the computed registration matrix was close to the unit matrix using a rigid transform. In spite of recent beam-forming correction research, the scan of a wide field of view is still a limit for current US technology due to dependency on frequency/axial resolution. No solution was proposed here for compensation of distortions or lateral resolution artifacts. Furthermore, in this work we used only US device and one transducer in 2D and 3D modes. More US devices and transducers could be tested to analyze the outcome and quantify the effect on the volume reconstruction.

In our analysis of the effects of the different parts of the transformation chain we found the error arising from the tracking system to be higher than the specified static error in dependence on the relative orientation of the tools mounted on the transducer and the phantom, respectively. The tracker error is a main contribution to the overall TRE and can hardly be minimized by optimally mounted tools due to limitations in a real-life interventional scenario.

The measured error of the US resolution was similar to the error specified by the producer. When using US calibration methods which focus on axial determination of targets, the higher lateral error might be of minor importance for the US calibration itself. Nevertheless the overall TRE is still in the magnitude of the lateral error.

For the US calibration we found the effect to the overall TRE to be much less than the theoretical upper bound, whereas for the US-US registration the error affected the overall TRE in a magnitude similar to its theoretical upper bound.

In this study a rigid transform was applied to perform the registration task: tests on a rigid body have demonstrated that *3D free-hand mode* and the *3D wobbler mode* can perform the intra-modal 3D-US/3D-US registration accurately. However, tests on deformable objects, such as prostates showed a higher error due to deformation, as expected. The 3D-3D/US-US registrations via *3D wobbler mode* on the egg shape rigid phantom (Table 4) showed a mean registration error of 0.82±0.44 mm, which is lower than the 2.67±1.46 mm mean error obtained from the registrations from prostate cases (Table 5). Nevertheless, the rigid registration provides a good initial alignment for a follow-up deformable registration [8]. Another potential source of error was given by the OTS: because some scanning orientations can compromise the tracking of the transducer position relative to the reference coordinate space, all tracking tools have to be clearly visible to the camera.

## 5 Conclusion

In this work we compared two 3D US modes to determine the most suitable one for 3D-3D US-US registration of the prostate. Both 3D methods showed comparable results with respect to localization and registration errors with phantom data. Our 3D-printed phantom has shown a high ability for fast and accurate 3D calibration while the simple pointer calibration technique was superior for 2D calibration. The tests on rigid and deformable bodies showed how image distortion, lateral resolution, motion and deformation artifacts, together with user ability, affect the 3D-3D registration task. The results indicate the different impact of each component of the chain to the final registration error which can be used for optimization purposes. The results indicated that the *3D wobbler mode* is a more feasible solution to limit these sources of errors than the *3D free-hand mode*.

## Appendix: Mathematical appendix

Pure translational error in one matrix

The undistorted vs. distorted chain from Eq (7)

$$F=M_{1}\ldots M_{k}\ldots M_{n}$$ (12)

F˜=M1…M˜k…Mn (13)

therefore we have

$$\begin{array}{c} F^{-1}\tilde{F}=M_{n}^{-1}\ldots M_{k-1}^{-1}M_{k}^{-1}\ldots M_{1}^{-1}M_{1}\ldots \tilde{M_{k}}M_{k+1}\ldots M_{n} \end{array}$$ (14)

$$\begin{array}{c} F^{-1}\tilde{F}=A^{-1}M_{k}^{-1}\tilde{M_{k}}A \end{array}$$ (15)

where $A=\left(\begin{array}{cc} R_{A} & T_{A}\\ 0 & 1 \end{array}\right)$ is a rigid body transformation. With $M_{k}=\left(\begin{array}{cc} R & T\\ 0 & 1 \end{array}\right)$, ${\tilde{M}}_{k}=\left(\begin{array}{cc} R & T+t\\ 0 & 1 \end{array}\right)$ we have $M_{k}^{-1}\tilde{M_{k}}=(\begin{array}{cc} I & R^{t}t\\ 0 & 1 \end{array})$ that is a purely translational error in *M*_*k*_ we have

$$\begin{array}{c} F^{-1}\tilde{F}=A^{-1}(\begin{array}{cc} I & R^{t}t\\ 0 & 1 \end{array})A=(\begin{array}{cc} I & R_{A}^{t}R^{t}t\\ 0 & 1 \end{array}) \end{array}$$ (16)

In summary, the effect of a translational error *t* in matrix *M*_*k*_ is a translational error of magnitude $\left|\right|R_{A}^{t}R^{t}t||=\left||t|\right|$ in the repositioning matrix.

Purely translational error in two matrices

In case of a translational error matrix appearing twice in the transformation chain, as is the case of the US calibration matrix, we have

$$\begin{array}{c} F^{-1}\tilde{F}=A^{-1}M_{k}^{-1}T{\tilde{M}}_{k}A \end{array}$$ (17)

where $A=\left(\begin{array}{cc} R_{A} & T_{A}\\ 0 & 1 \end{array}\right)$ and $T=\left(\begin{array}{cc} I & t\\ 0 & 1 \end{array}\right)$, *M*_*k*_ is US calibration matrix. With $M_{k}=\left(\begin{array}{cc} R & T\\ 0 & 1 \end{array}\right)$ we have

$$\begin{array}{c} F^{-1}\tilde{F}=A^{-1}M_{k}^{-1}T{\tilde{M}}_{k}A=(\begin{array}{cc} I & R_{A}^{t}\left(R^{t}t-t\right)\\ 0 & 1 \end{array}) \end{array}$$ (18)

In summary, the effect of an translational error *t* in the US calibration matrix is a translational error of magnitude $\left|\right|R_{A}^{t}\left(R^{t}-I\right)t||=\left|\right|\left(R^{t}-I\right)t||\leq 2||t||$ in the repositioning matrix, where *R* is the rotational part of the US-calibration matrix.

## Supporting information

S1 Data (ZIP)
