# Supplementary figures and images for: Evaluation of 3D ultrasound for image guidance
Source: PLoS One. 2020 Mar 26;15(3):e0229441. doi: 10.1371/journal.pone.0229441 (PMC7098612; doi:10.1371/journal.pone.0229441)

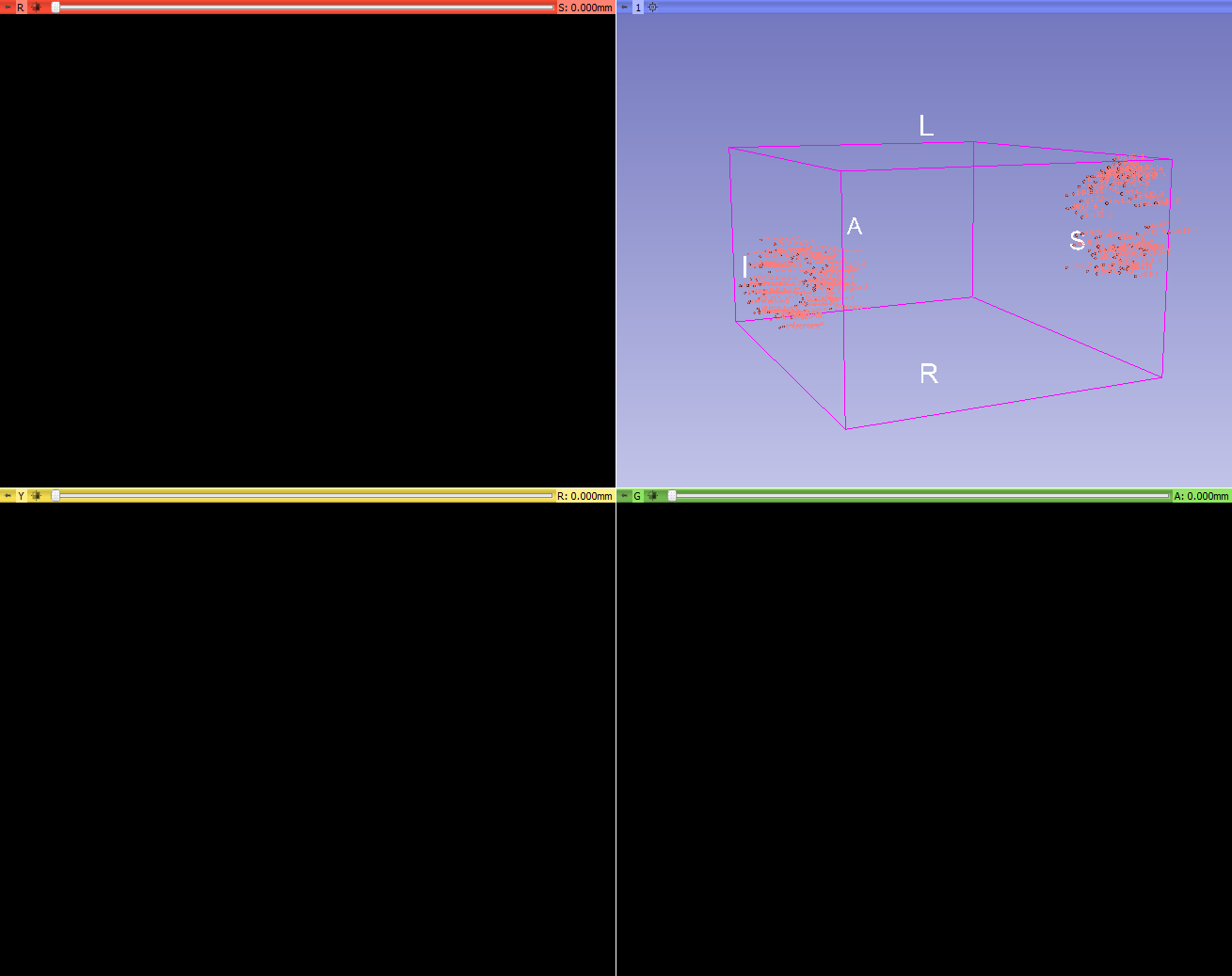

Supplement: S1 Data — (ZIP) [file pone.0229441.s001.zip › Data_PLOS_ONE - light/1_Calibratation error analyisis/Calibration - 3D wobbler/Pointtopoint/Total scene/Master Scene View.png]

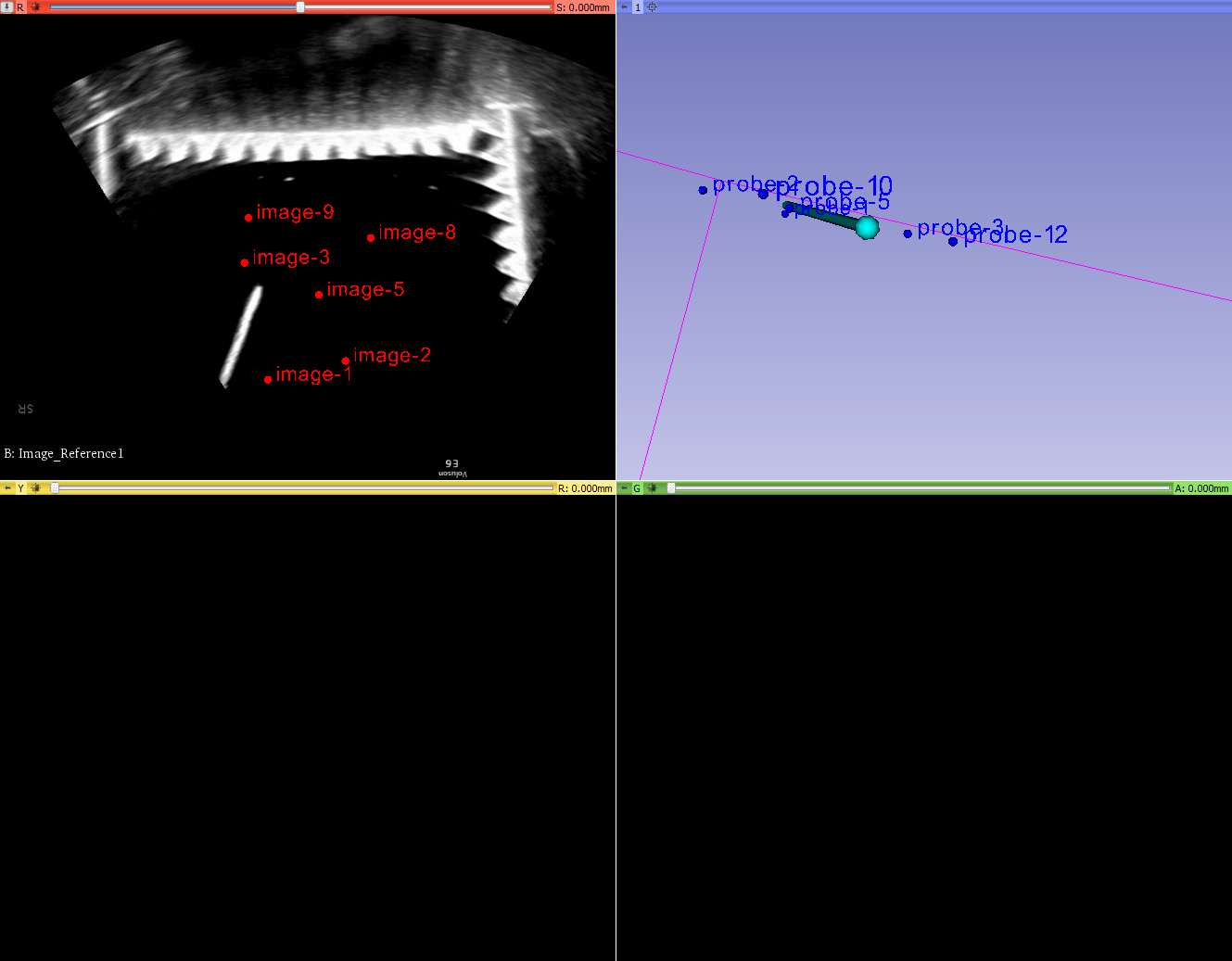

Supplement: S1 Data — (ZIP) [file pone.0229441.s001.zip › Data_PLOS_ONE - light/1_Calibratation error analyisis/Calibration -3D freehand/pointer/15 cm 2D/Master Scene View.png]

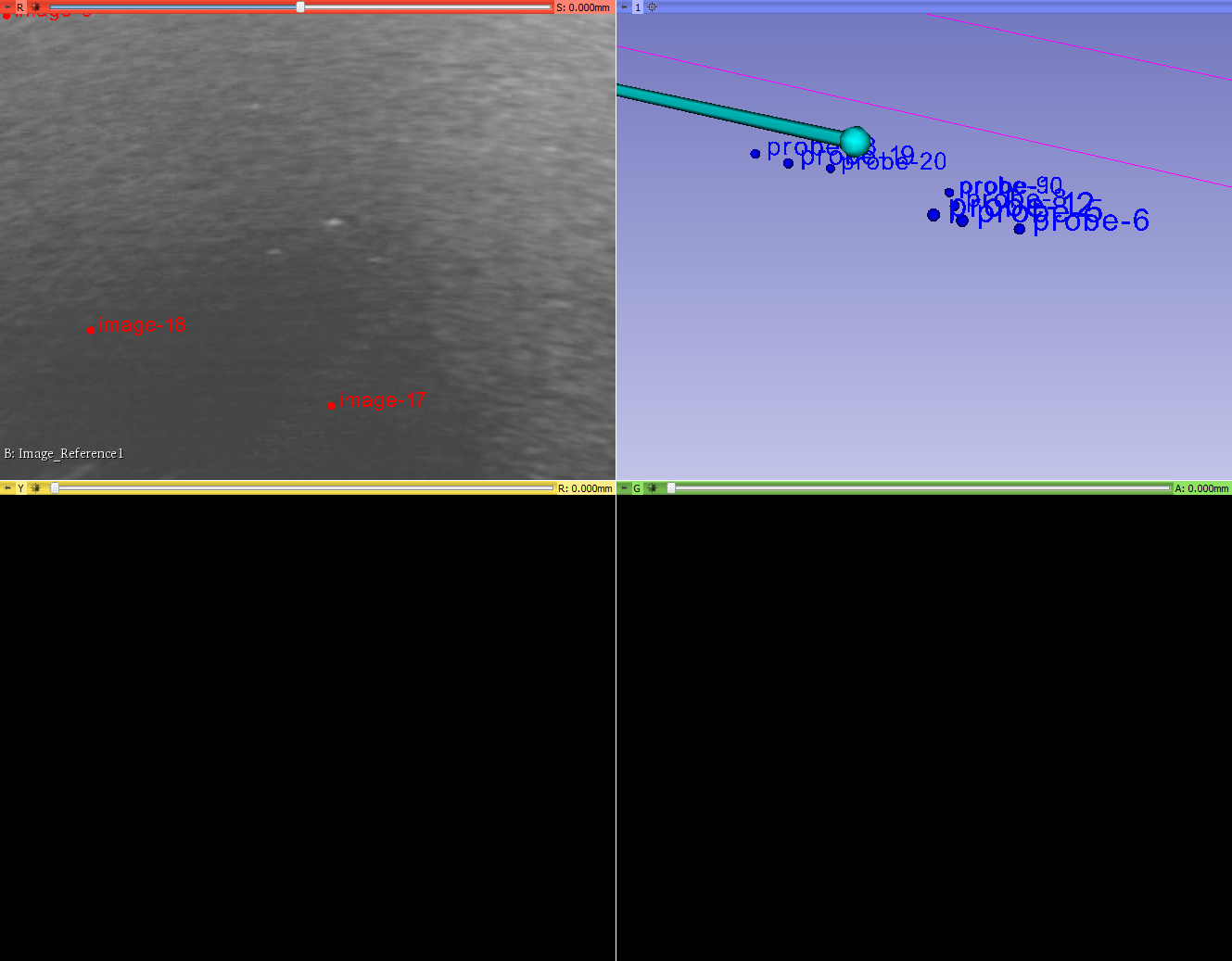

Supplement: S1 Data — (ZIP) [file pone.0229441.s001.zip › Data_PLOS_ONE - light/1_Calibratation error analyisis/Calibration -3D freehand/pointer/7.4 cm 2D/Master Scene View.png]

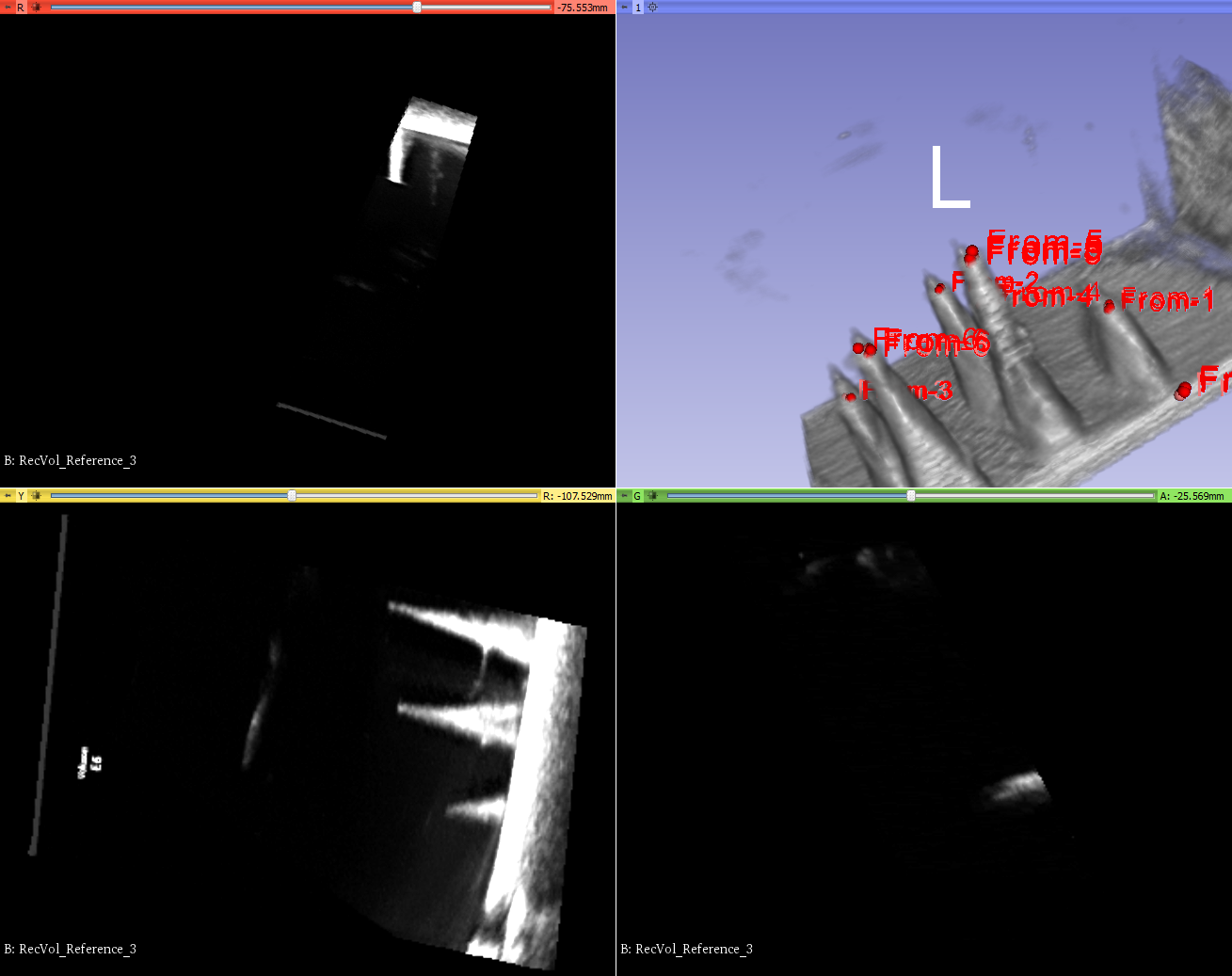

Supplement: S1 Data — (ZIP) [file pone.0229441.s001.zip › Data_PLOS_ONE - light/1_Calibratation error analyisis/Target error - 3D freehand/15cm/sweep pointer/Master Scene View.png]

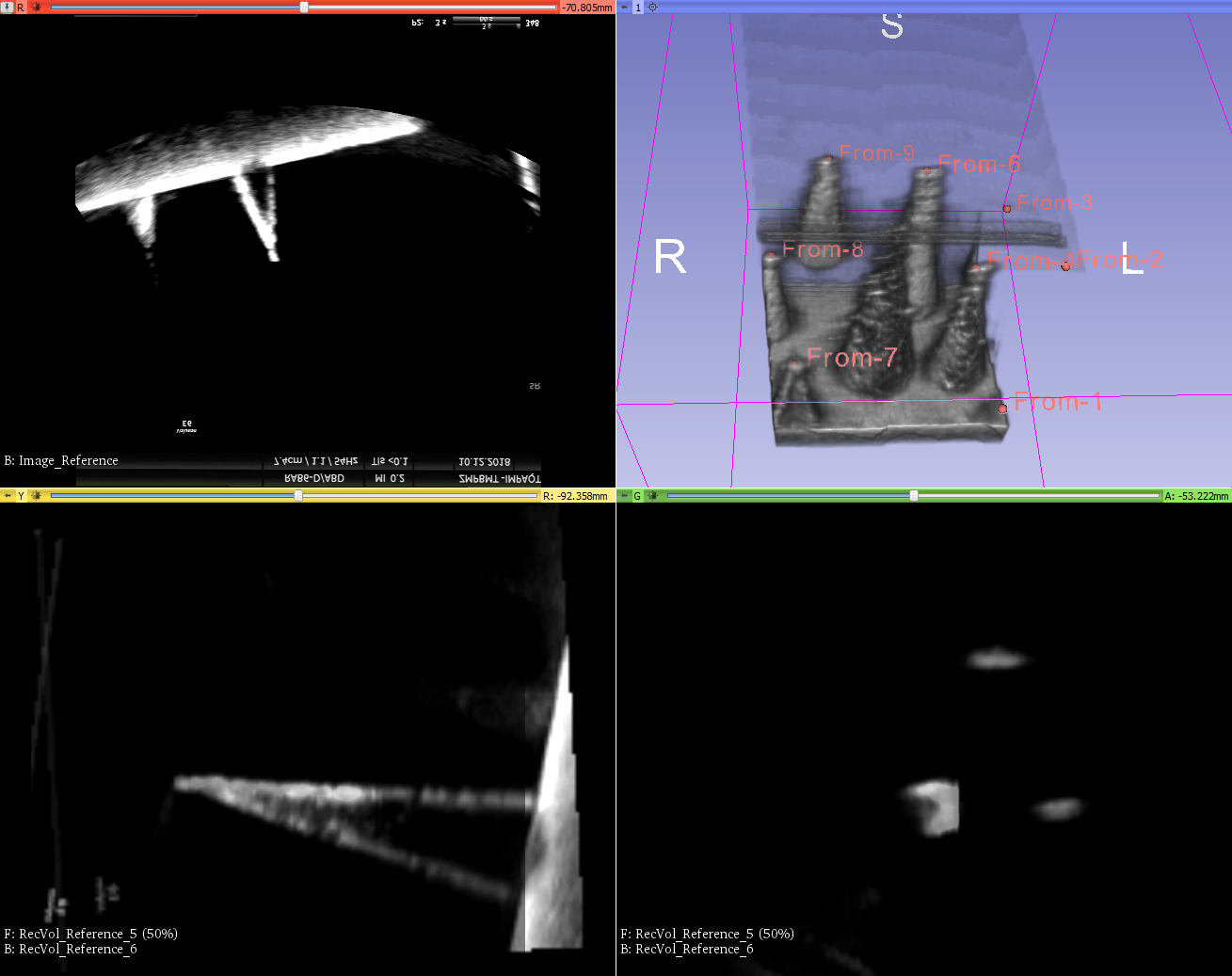

Supplement: S1 Data — (ZIP) [file pone.0229441.s001.zip › Data_PLOS_ONE - light/1_Calibratation error analyisis/Target error - 3D freehand/7.4cm/sweep Nwire/Master Scene View.png]

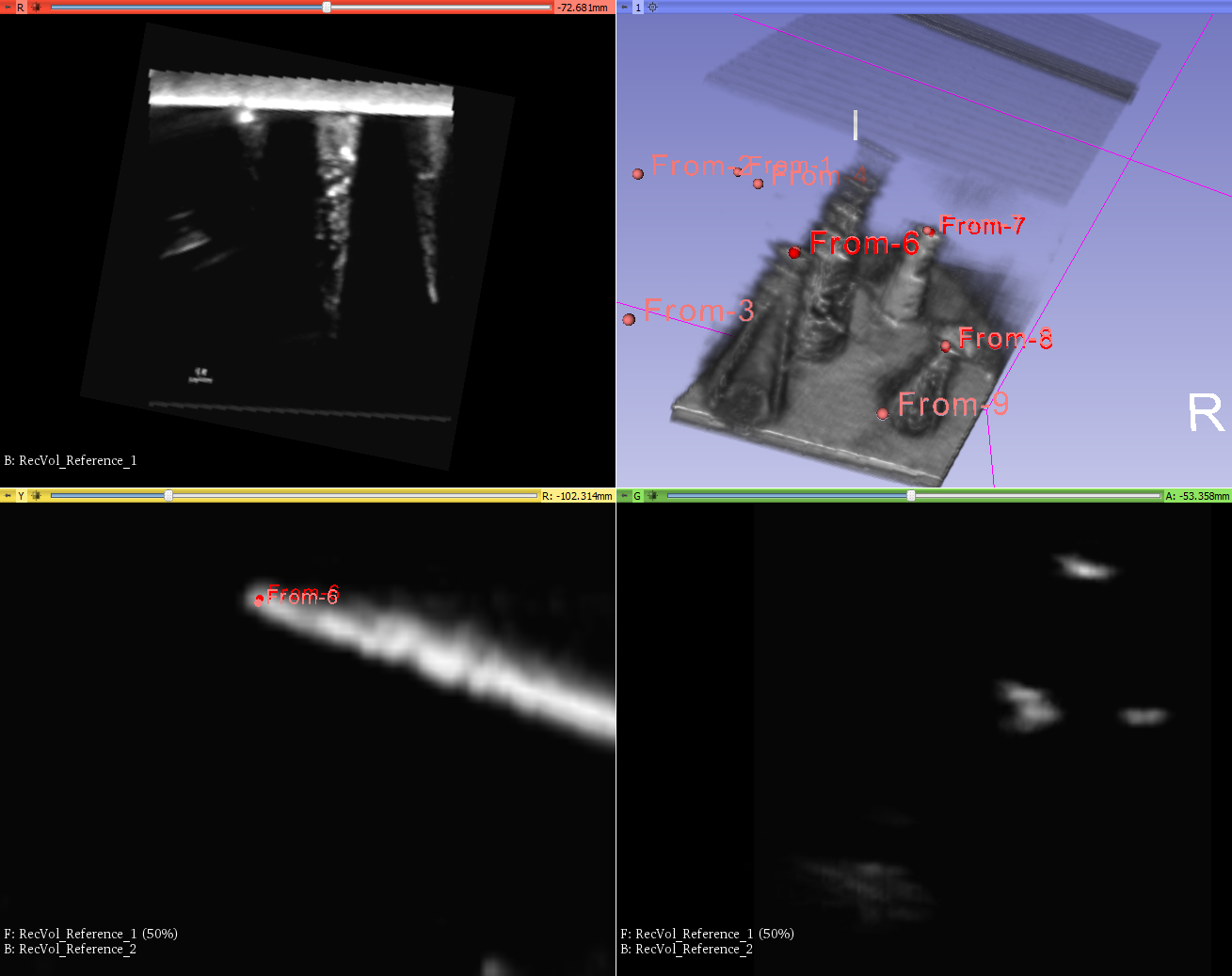

Supplement: S1 Data — (ZIP) [file pone.0229441.s001.zip › Data_PLOS_ONE - light/1_Calibratation error analyisis/Target error - 3D freehand/7.4cm/sweep pointer/Master Scene View.png]

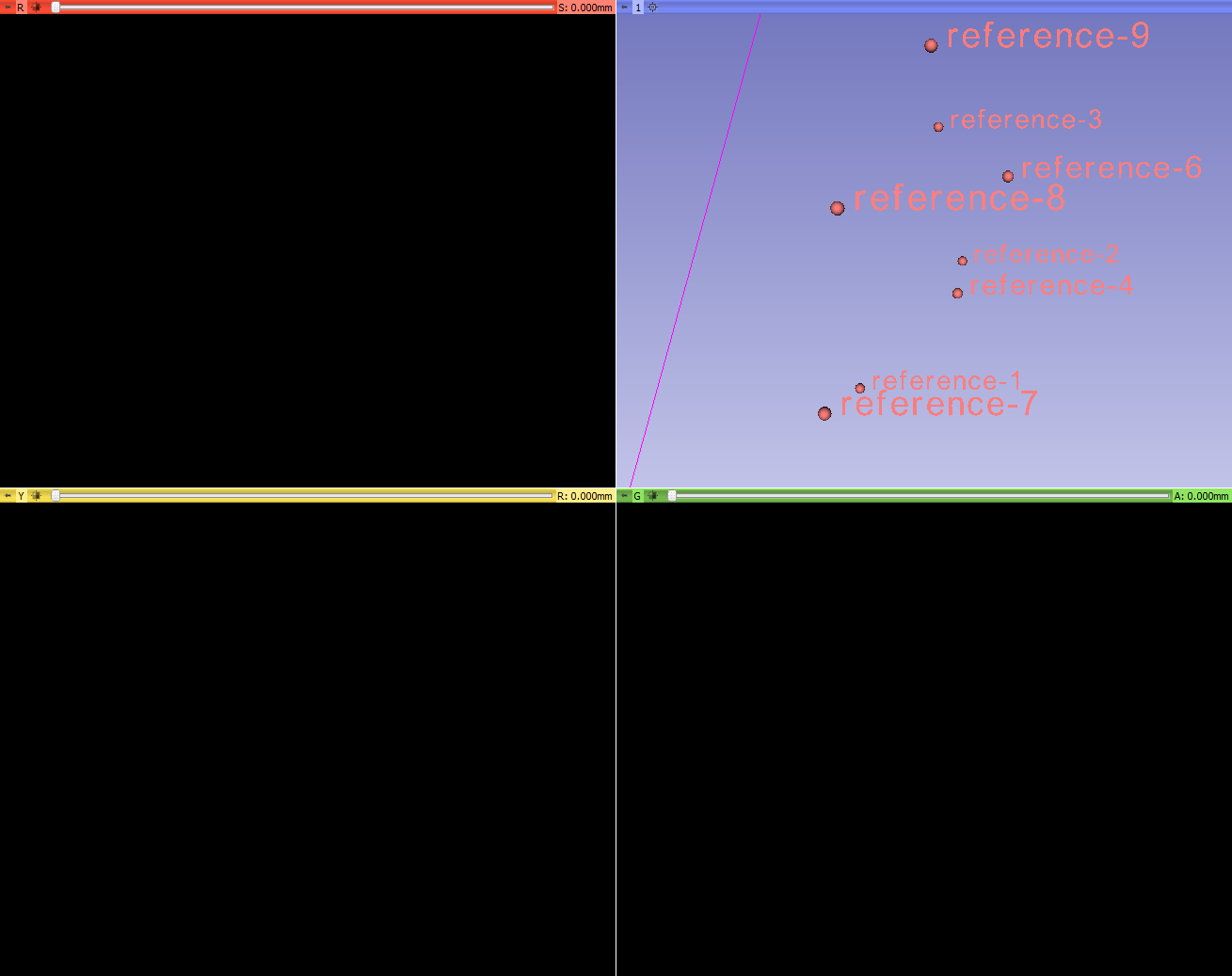

Supplement: S1 Data — (ZIP) [file pone.0229441.s001.zip › Data_PLOS_ONE - light/1_Calibratation error analyisis/Target error - 3D wobbler/7.4cm/reference points phantom cones/2nd/Master Scene View.png]
